# 15例大颗粒淋巴细胞白血病临床特征单中心研究

**DOI:** 10.3760/cma.j.cn121090-20250407-00165

**Published:** 2026-03

**Authors:** 芯 谢, 荷莲 李, 倩 王, 菊 李, 林 刘, 利 王, 小华 罗

**Affiliations:** 1 重庆医科大学附属第一医院血液内科，重庆 400016 Department of Hematology, the First Affiliated Hospital of Chongqing Medical University, Chongqing 400016, China; 2 重庆市九龙坡区人民医院肾病风湿免疫科，重庆 400050 Department of Nephrology, Rheumatology and Immunology, Jiulongpo District People's Hospital of Chongqing, Chongqing 400050, China; 3 陆军军医大学第一附属医院血液内科，重庆 400038 Department of Hematology, the First Affiliated Hospital of Army Medical University, Chongqing 400038, China

## Abstract

回顾性分析2017年1月至2024年12月于重庆医科大学附属第一医院诊断的15例大颗粒淋巴细胞白血病（LGLL）患者的临床资料。患者中位年龄50岁，主要表现为乏力（73.3％）、脾肿大（46.7％）、淋巴结肿大（33.3％），均未合并自身免疫性疾病。实验室检查主要表现为血红蛋白水平下降（100％）和（或）中性粒细胞减少（73.3％），骨髓涂片可见淋巴细胞比例升高，5例合并纯红细胞再生障碍，常见免疫表型为TCRαβ^+^TCRγδ^-^CD3^+^CD4^-^CD8^+^CD16^-^CD10^-^CD30^-^CD45RA^+^CD45RO^-^CD56^-^CD25^-^，所有患者均可见T细胞单克隆增殖证据，8例患者存在补体C3或C4下降。一线免疫抑制治疗总有效率为85.7％（12/14）。

## Abstract (en)

This study retrospectively analyzed the clinical data of 15 patients diagnosed with large granular lymphocytic leukemia（LGLL）at the First Affiliated Hospital of Chongqing Medical University between January 2017 and December 2024. The median age was 50 years, and the main clinical manifestations included fatigue（73.3％）, splenomegaly（46.7％）, and lymphadenopathy（33.3％）. None of the patients had autoimmune diseases. Laboratory examinations primarily demonstrated decreased hemoglobin levels（100％）and/or neutropenia（73.3％）. The bone marrow smears showed an increase in lymphocyte proportion, and pure red cell aplasia was frequently observed. The typical immunophenotype was TCRαβ^+^TCRγδ^-^CD3^+^CD4^-^CD8^+^CD16^-^CD10^-^CD30^-^CD45RA^+^CD45RO^-^CD56^-^CD25^-^, with monoclonal T-cell proliferation confirmed in all patients. Further, 8 patients demonstrated decreased complement C3 or C4 levels. The overall response rate of first-line immunosuppressive therapy for patients with LGLL was 85.7％（12/14）.

大颗粒淋巴细胞白血病（Large granular lymphocytic leukemia, LGLL）是一组罕见的白血病，根据WHO第五版造血与淋巴组织肿瘤分类[1]，其属于成熟T细胞和NK细胞白血病，包括T大颗粒淋巴细胞白血病（T-LGLL）和NK大颗粒淋巴细胞白血病（NK-LGLL）。LGLL特征是含粗大嗜天青颗粒的淋巴细胞的持续性克隆性增殖（时间>6个月），且没有明确的原因[2]。LGLL常为惰性表现，但部分恶性程度高，临床进展迅速且预后较差。本研究旨在通过回顾性分析中国西南地区单中心15例LGLL患者的临床特征及诊疗经过以加深对该疾病的认识。

## 病例与方法

1. 病例选择及一般资料：收集2017年1月至2024年12月重庆医科大学附属第一医院诊断的15例LGLL患者的临床资料进行回顾性分析，包括一般特征、主要症状、血常规检查、免疫学检查、骨髓检查、流式细胞术免疫分型、细胞遗传学及分子生物学分析、治疗方案，以及预后等。

2. 诊断标准和疗效评价：LGLL诊断标准[1],[3]具体包括血细胞减少相关临床表现、外周血大颗粒淋巴细胞（LGL）增多、骨髓或外周血流式细胞术检测可见特征性免疫表型以及TCR基因重排或其他方法证实的T/NK细胞克隆性增殖，STAT3或STAT5B突变也有助于诊断。LGLL患者在持续治疗4个月后评估治疗反应[4]。完全缓解（CR）：外周血中性粒细胞>1.5×10^9^/L、HGB>110 g/L、PLT>100×10^9^/L、淋巴细胞绝对值<4×10^9^/L且LGL计数处于正常值范围［（0.2～0.4）×10^9^/L］，维持至少3个月；部分缓解（PR）：血细胞计数缓解但未达到上述标准；治疗失败（NR）：未能达到CR或PR；疾病复发：疾病缓解后血红蛋白显著下降，需要输血治疗或恢复足量药物治疗。

3. 统计学处理：采用SPSS 27.0对数据进行统计学分析，连续数据以中位数（*IQR*）描述，分类数据以比例（％）描述。

## 结果

1. 基本资料：收集15例LGLL患者的临床资料，详见表1，15例均为T-LGLL患者；男8例，女7例；中位年龄50（范围：27～86）岁。12例患者初诊时有临床症状，11例（73.3％）患者出现乏力或头昏，活动力较前下降，1例（6.7％）患者有出血情况，为齿龈出血；3例（20.0％）为体检时发现贫血或血细胞减少。7例（46.7％）患者初诊时有脾肿大；5例（33.3％）患者有淋巴结肿大；所有患者均无肝肿大。15例患者初诊时均有血常规异常，以贫血、中性粒细胞减少、淋巴细胞增多为主。15例患者外周血LGL绝对值中位数1.11（0.55，2.42）×10^9^/L，外周血细胞计数见表1。15例患者均有贫血，其中1例轻度贫血，4例中度贫血，10例重度贫血；8例患者白细胞减少，1例患者白细胞增多，11例患者中性粒细胞减少；12例患者淋巴细胞比例升高；3例患者血小板减少。15例患者均行骨髓穿刺检查。5例患者骨髓象提示为纯红细胞再生障碍性贫血（PRCA），2例患者红系增生减低；10例骨髓可见淋巴细胞数量增多，1例患者提示大颗粒淋巴细胞增多。

**表1:** 15例大颗粒淋巴细胞白血病患者的临床特征及实验室检查结果

特征	数值
性别（例，男/女）	8/7
年龄［岁，*M*（范围）］	50（27～86）
乏力/头昏［例（％）］	11（73.3）
出血［例（％）］	1（6.7）
脾肿大［例（％）］	7（46.7）
淋巴结肿大［例（％）］	5（33.3）
LGL绝对值［×10^9^/L，*M*（*IQR*）］	1.11（0.55，2.42）
LGL>2×10^9^/L［例（％）］	5（33.3）
LGL（0.5～2）×10^9^/L［例（％）］	7（46.7）
LGL<0.5×10^9^/L［例（％）］	3（20.0）
WBC［×10^9^/L，*M*（*IQR*）］	3.40（2.15，6.26）
中性粒细胞绝对计数［×10^9^/L，*M*（*IQR*）］	1.44（0.37，1.53）
中性粒细胞比例［％，*M*（*IQR*）］	24.7（11.6，48.0）
淋巴细胞绝对计数［×10^9^/L，*M*（*IQR*）］	1.39（0.79，4.42）
淋巴细胞比例［％，*M*（*IQR*）］	60.0（22.2，73.6）
HGB［g/L，*M*（*IQR*）］	52（47，66）
RBC［×10^12^/L，*M*（*IQR*）］	1.66（1.30，1.89）
PLT［×10^9^/L，*M*（*IQR*）］	198（101，288）
网织红细胞绝对值［×10^9^/L，*M*（*IQR*）］	19.3（8.6，43.5）
网织红细胞比例［％，*M*（*IQR*）］	1.5（0.5，2.0）
疾病分型［例（％）］	
T-LGLL-TCRαβ	12（80.0）
T-LGLL-TCRγδ	3（20.0）

**注** LGL：大颗粒淋巴细胞；T-LGLL：T大颗粒淋巴细胞白血病

2. 实验室检查：15例患者均进行了流式细胞术检测（表2），显示其常见免疫表型为TCRαβ^+^TCRγδ^-^CD3^+^CD4^-^CD8^+^CD16^-^CD10^-^CD30^-^CD45RA^+^CD45RO^-^CD56^-^CD25^-^。15例患者至少完善TCR基因重排、TCRVβ、TRBC1其中一项检测，结果均提示存在T细胞单克隆。9例患者行TCR基因重排检测，TCRβ检出率100％（9/9），TCRγ检出率88.9％（8/9），TCRδ检出率33.3％（3/9），TCRβ、TCRγ、TCRδ同时检出率33.3％（3/9）。7例患者完善流式细胞术TCRVβ检测，均提示单克隆，3例TCRVβ24个亚单位之和小于20％，4例分别单克隆表达TCRVβ4、TCRVβ5.3、TCRVβ11和TCRVβ20。5例患者行流式细胞术TRBC1检测，4例不表达TRBC1，1例单克隆表达TRBC1。2例患者同时完善流式细胞术TCRVβ、TRBC1检测，结果一致均提示单克隆。4例患者完善基因二代测序，1例患者为BCOR基因突变，1例为STAT3、TET2、DNMT3A、GATA2突变，1例为FLT3、ASXL2、KMT2C突变，1例未检测到基因突变。

**表2:** 15例大颗粒淋巴细胞白血病患者的骨髓流式细胞术免疫分型结果

抗原	例1	例2	例3	例4	例5	例6	例7	例8	例9	例10	例11	例12	例13	例14	例15	表达率（％）
CD2	+	dim	+	+	+	+	dim	+	+	+	+	dim	+	+	+	100
CD7	+	dim	p	−	−	+	+	+	+	+	+	+	+	−	+	80.0
CD5	+	dim	dim	−	dim	+	+	+	−	+	+	+	+	+	−	80.0
CD3	bri	bri	bri	+	dim	+	+	+	+	+	+	+	bri	+	+	100
CD4	−	−	−	−	−	+	−	−	−	dim	+	−	−	−	−	20.0
CD8	+	bri	+	+	dim	+	+	+	+	+	+	+	p	p	−	93.3
TCRαβ	+	+	+	+	dim	/	+	/	/	/	/	+	/	−	/	87.5
TCRγδ	−	/	−	−	−	/	−	/	−	−	/	−	+	+	+	27.3
TCRγδ1	/	/	/	−	−	/	/	/	−	−	/	−	−	+	+	25.0
TCRγδ2	/	/	/	−	−	/	/	/	−	−	/	−	−	−	−	0
CD45RA	+	+	+	+	+	/	+	/	/	/	/	+	+	/	+	100
CD45RO	−	−	p	−	−	/	−	/	−	dim	/	−	/	/	/	22.2
CD10	−	−	−	−	−	/	−	−	−	−	/	−	−	−	−	0
CD16	/	/	−	−	−	−	−	−	−	−	/	−	−	−	/	0
CD56	/	−	−	−	−	−	−	−	+	/	/	−	+	−	p	25.0
CD57	−	+	p	p	p	/	+	p	p	p	p	p	−	p	/	84.6
CD30	−	/	−	−	−	/	−	−	−	−	/	−	−	−	/	0
CD25	−	−	−	−	−	/	+	/	−	−	/	−	−	−	−	8.3

**注** +：阳性表达（胞膜或胞质抗原表达比例≥80％）；p：部分阳性表达（胞膜抗原表达比例20％～80％，胞质抗原10％～80％）；−：阴性表达（胞膜抗原表达比例<20％，胞质<10％）；bri：强表达；dim：弱表达；/：未检测

5例患者行类风湿因子检测，其中1例患者类风湿因子升高，为260 IU/ml。12例患者进行ANA抗体测定，其中2例低滴度阳性，为1∶100。11例患者进行ANCA谱抗体测定，均为阴性。10例患者进行体液免疫检查，7例患者补体C3下降，其中1例患者同时伴免疫球蛋白M升高，1例患者伴补体C4下降；另外1例患者仅免疫球蛋白G下降，1例患者仅补体C4下降，1例患者未见明显异常。13例患者行EBV-DNA病毒载量检测，3例患者检测到EB病毒复制，病毒载量分别为2.97×10^3^拷贝/ml、3.65×10^3^拷贝/ml、5.04×10^2^拷贝/ml，前1例患者多次随访病毒载量转阴，后2例患者未随访病毒载量。13例患者行CMV-DNA病毒载量检测，3例患者行HTLV-DNA病毒载量检测，均未检测到病毒复制。

3. 治疗方案及治疗反应：15例患者中除1例患者无治疗指征，其余14例患者均接受治疗，一线治疗总反应率为85.7％。11例患者一线治疗方案为环孢素（CsA）单药治疗，持续治疗4个月时总反应率为81.8％，CR 1例（9.1％），PR 8例（72.7％）。2例CsA治疗NR的患者二线治疗方案予以甲氨蝶呤（MTX），治疗反应仍为NR，其中1例先后予以抗胸腺球蛋白（ATG）、硼替佐米、环磷酰胺（CTX），均为NR，后再次调整为CsA，治疗反应为PR；另1例三线治疗方案予以环磷酰胺联合泼尼松治疗，治疗反应为PR，因毛囊炎停用泼尼松后，血红蛋白进行性下降至32 g/L，后调整为西罗莫司联合罗沙司他，治疗反应达PR后，患者出现口腔溃疡，调整为单药西罗莫司治疗，治疗反应为PR。非CsA单药治疗中1例患者一线治疗方案为MTX联合泼尼松，治疗反应为PR；1例患者接受CsA联合泼尼松，治疗持续4个月时治疗反应为PR，治疗3年后达到CR，治疗约5年后复查血红蛋白下降，提示复发，予以氟达拉滨+环磷酰胺（FC）方案化疗3个疗程后，达CR，现予以MTX维持治疗；1例患者接受ATG治疗，治疗反应为CR。

## 讨论

LGLL是一种T细胞或NK细胞克隆性增殖的一组疾病，临床上以贫血、中性粒细胞减少有关的反复感染、脾大等为主要表现。LGLL的诊断核心在于找到大颗粒T/NK淋巴细胞的克隆性增殖证据[5]，临床上通过TCR基因重排检测、流式细胞术TCRVβ和（或）TRBC1检测证明T细胞的克隆性，是T-LGLL的主要诊断依据。

本研究患者就诊时中位年龄为50（27～86）岁，与国外既往研究报道[6]–[7]相比，发病年龄更年轻，但更接近国内既往报道的病例研究[8]–[10]。在本研究中，40.0％（6/15）的患者低于50岁，50～60岁年龄段例数最多，占40.0％（6/15）。本研究的发病特点可能与以下原因有关：有症状的患者较多，本组80.0％的患者就诊时有症状；种族因素或遗传因素与国外的差异，国外以中性粒细胞下降导致的反复感染为主要表现[11]，国内以贫血导致的乏力、头昏等为主要表现[12]–[13]。LGLL的临床特征需要国内更大规模的流行病学调查进一步证实。

国外研究表明，15％～40％的LGLL患者合并自身免疫性疾病[14]，而与既往国内研究类似[8],[10]，本组患者均未合并自身免疫性疾病，但8例患者存在补体C3或C4下降，提示仍存在补体系统异常。Gentile等[15]报道T-LGLL患者存在多种体液免疫异常，其中72％的患者有免疫复合物形成。免疫复合物形成通过经典途径激活补体系统，启动补体级联反应，C3被裂解为C3a和C3b[16]–[17]，可导致血浆中补体C3下降。Lee等[18]的实验表明STAT3 Y705磷酸化是由补体激活介导的。Teramo等[19]研究证实STAT3的过表达和激活是所有LGLL患者的共同特征，JAK2/STAT3通路激活有助于维持LGL的存活。因此，我们认为国内T-LGLL虽然自身免疫性疾病发生率不高，但是仍存在体液免疫异常，并且补体系统激活介导STAT3磷酸化，可能与T-LGLL的发生机制有关。

国际上T-LGLL经典免疫表型为TCRαβ^+^CD3^+^CD4^-^CD5^dim^CD8^+^CD16^+^CD45RO^-^CD45RA^+^CD57^+^[11],[20]，这与国内大部分病例报道并不一致。国内董玉婷等[21]报道21例T-LGLL患者中40％表达CD16，朱阳敏等[8]报道108例T-LGLL患者中2％表达CD16，本研究15例T-LGLL患者均不表达CD16。STAT3突变的CD8^+^CD4^-^T-LGLL患者中97.3％表达CD16，而STAT3野生型的CD8^+^CD4^-^T-LGLL患者中73.4％不表达CD16[22]；研究发现T-LGLL的罕见侵袭性形式通常携带STAT5b突变[23]，其典型特征表现为CD8^+^CD56^+^CD16^-^CD57^-^表型的大颗粒淋巴细胞增殖，这提示CD16的表达可能与STAT3、STAT5b突变有关。STAT3突变与临床表现更严重有关[22]，如中性粒细胞减少症发生率更高、伴随类风湿性关节炎等自身免疫性疾病、更频繁的治疗需求以及更短的总生存期。Barilà等[24]在研究中也发现了LGL表型和STAT3状态的联系，CD8^+^CD16^+^CD56^-^表型的T-LGLL患者STAT3突变和中性粒细胞减少发生率更高，而CD4^+^CD8^±^ T-LGLL患者常为STAT5b突变，无STAT3突变。CD16表达似乎在γδ T-LGLL中更常见，Teramo等[25]报道36例γδ T-LGLL患者CD16表达率为92％；Barilà等[26]报道γδ T-LGLL的CD16表达率高于αβ T-LGLL（72.3％对45.7％，*P*<0.001）。本研究中仅4例患者进行二代基因测序，其中1例患者有STAT3突变，其属于CD8^+^CD4^-^CD16^-^ γδ T-LGLL，限于本中心病例数量及行NGS检测患者较少，CD16在T-LGLL患者中的表达有待进一步研究。

近年来，随着精准检测技术的发展，NGS和单细胞测序在LGLL诊疗中应用逐渐增多。Barilà等[24]报道205例LGLL患者，发现约40％的患者发生体细胞STAT3和STAT5b突变；Huuhtanen等[27]利用单细胞转录组测序，进一步揭示了T-LGLL细胞比健康反应性T细胞有更强的细胞毒性和耗竭性。NGS检测、单细胞测序在LGLL的诊断、发病机制和靶向治疗方面提供了全面的分子视角，未来在临床实践中，应积极推广应用，以期获得个体化治疗。

LGLL目前多采用以CsA、MTX、环磷酰胺为基础的免疫抑制治疗，联合或不联合激素。本组患者主要以CsA治疗为主，虽然治疗的总缓解率尚可，但CR率偏低，因此迫切需要寻找新型药物改善这一状况。近期，国内一项多中心、随机Ⅱ期临床试验[28]评估了TPM（沙利度胺联合泼尼松、MTX）方案在LGLL患者中的疗效，CR率可达75.0％，有望进一步推广于临床。

总之，本研究显示LGLL患者年龄较国外更为年轻，主要分布于40～60岁，可能存在地区差异；补体C3或者C4在大多数患者中下降，虽然没有患者合并自身免疫性疾病，但仍需要考虑LGLL患者存在免疫异常现象。本研究中LGLL患者的免疫表型与既往研究并不完全一样，均不表达CD16。当前LGLL经验性治疗CR率仍然偏低，亟待更多新的治疗方案以进一步改善预后。
